# The impact of active live-stream participation on wellbeing: the mediating roles of social presence and connectedness and the moderating role of fear of missing out

**DOI:** 10.3389/fpsyg.2026.1824481

**Published:** 2026-08-21

**Authors:** Peilu Qin, Yujia Lin

**Affiliations:** 1 College of Communication Science and Art, Chengdu University of Technology, Chengdu, Sichuan, China; 2 School of Journalism and Communication, Sichuan International Studies University, Chongqing, China

**Keywords:** active participation, fear of missing out, live streaming, social connectedness, social presence, subjective wellbeing

## Abstract

**Background:**

Chat-based live streaming is a highly interactive digital environment in which viewers comment, react, and engage in real time. However, the psychological mechanisms and boundary conditions linking active participation to wellbeing remain unclear. Drawing on self-determination theory and social presence theory, this study examined whether social presence and social connectedness sequentially mediate the association between active live-stream participation and subjective wellbeing, and whether trait fear of missing out (FoMO) moderates the participation-based pathways.

**Methods:**

A cross-sectional online survey was conducted with 467 adult viewers of chat-based live-streaming platforms in China. A regression-based moderated serial mediation model, corresponding to conceptual Model 85, was estimated in Python. The analyses controlled for 11 demographic and behavioral covariates and used heteroscedasticity-consistent standard errors. Johnson–Neyman analyses were used to probe conditional associations across levels of FoMO.

**Results:**

Active participation was positively associated with subjective wellbeing, both directly and indirectly through social presence and social connectedness, independently and in sequence. Trait FoMO was essentially unrelated to wellbeing at the bivariate level but robustly moderated the participation-based pathways. Johnson–Neyman analyses indicated that participation was positively associated with wellbeing for roughly three quarters of viewers, specifically those below a FoMO level of 3.40 on the 1–5 scale. This association weakened to non-significance at higher levels of FoMO and reversed only among a small minority of viewers, approximately 7%, at the highest levels of FoMO.

**Conclusion:**

Active participation in chat-based live streaming may function as a relational resource associated with subjective wellbeing, but its psychological value is conditional on dispositional anxiety. FoMO operated in this study as a boundary condition on the quality of participation rather than as a direct determinant of flourishing.

## Introduction

Live streaming is an emerging form of online social media that has attracted large numbers of users because of its high immediacy and interactivity (Xue et al., 2020). The popularity of live-streaming services has transformed online entertainment from an asynchronous consumption model into highly synchronous and interactive social practices. Whereas, traditional video-on-demand offers asynchronous viewing, live streaming features impromptu content and transient experiences in which streamers and viewers occupy the same temporal space. Viewers are no longer passive bystanders; they actively participate through real-time comments, virtual gifts, and continuous viewing, co-constructing a shared sense of reality. Prior research indicates that this engaging interaction partially satisfies basic psychological needs for connection and cognition (Shen, 2021). At the same time, live-streaming participation raises complex questions about the quality of digital connection.

Existing empirical work presents divided conclusions. On the one hand, high participation may promote social connection, facilitate social capital accumulation, and alleviate loneliness (Goh and Tandoc, 2024; Roberts and David, 2020). On the other hand, anxiety may drive participation into compulsive consumption (Chen and Chang, 2019), particularly under scarcity persuasion and fear of missing out, where hedonic motives are continuously amplified, potentially producing a contradictory experience of later regret amid in-the-moment enjoyment (Roostika and Aji, 2025) or even problematic use (Wang and Wang, 2024). It is therefore important to clarify when participation is associated with psychological resources and when it is more closely tied to anxiety-driven compensatory experiences.

Within the live-streaming ecosystem, chat-based live streaming is a rapidly developing sub-genre with distinctive psychological characteristics. Unlike game or performance streaming, which rely on professional skills or structured formats, chat-based streaming features low participation barriers and centers on emotional companionship. Streamers often act as digital companions, building immediate intimacy through fluid, unstructured conversation, while viewers respond through likes, comments, and virtual gifts (Yang et al., 2023). Interactive content attracts continuous viewing and is associated with pleasant experiences (Chen and Lin, 2018; Shen, 2021; Xi et al., 2023), and immersed viewers are more likely to engage in continuous active participation (Gong et al., 2020; Joo and Yang, 2023). Because chat-based streams often remain open without clear ending points, they create a psychological context in which participation may relate to relational resources through immediate emotional companionship, or, alternatively, in which the pressure of continuous online presence and fear of missing out may render participation an anxiety-driven experience.

The present study focuses on subjective wellbeing as the outcome of interest. Subjective wellbeing is a multidimensional construct encompassing hedonic experiences centered on pleasure and eudaimonic dimensions centered on self-realization and meaning (Ryff and Keyes, 1995; Zhang and Xiao, 2025), reflecting autonomy, environmental mastery, personal growth, positive relations, purpose, and self-acceptance (Ryff and Singer, 2008; Seligman, 2018). Importantly, contemporary models conceptualize positive mental health (flourishing) and mental illness (distress) as related but distinct continua rather than opposite poles of a single dimension (Keyes, 2005); the present study indexes the positive, flourishing continuum. From a self-determination perspective, wellbeing depends heavily on the satisfaction of basic psychological needs, particularly the need for relatedness, the degree to which an individual feels accepted, understood, and cared for (Deci et al., 2017). In the live-streaming context, real-time synchronization and interactivity may prompt participants to experience pleasant states theorized to relate to life satisfaction. Although prior work has examined live streaming and wellbeing across e-commerce (Lin et al., 2026; Roostika and Aji, 2025), tourism (Zhang and Xiao, 2025), gaming (Firayani, 2025), and live-streamed instruction (Lei et al., 2026; Liu et al., 2021), research on chat-based live streaming remains limited.

Because chat-based live streaming lacks clear termination cues, trait fear of missing out (FoMO) is a theoretically critical boundary condition. FoMO is a relatively stable disposition characterized by persistent worry about missing rewarding or important experiences that others are having (Przybylski et al., 2013). It is closely tied to heightened sensitivity to social-connection cues and to a behavioral pattern of staying constantly online to monitor the social environment (Vanden Abeele and van Rooij, 2016; Beyens et al., 2016). A frustrated need to belong is considered a motivational root of FoMO, which has been associated with elevated anxiety and lower wellbeing (Baker et al., 2016; Wolniewicz et al., 2018). For individuals high in FoMO, the social cues that ordinarily support social presence may not translate into secure or intimate experiences; instead, they may intensify vigilance and anxiety about missing upcoming interactions, occupying psychological resources needed to process social cues and construct relational meaning (Biocca et al., 2003; Lowry et al., 2009). Whether participation translates into relational benefits, then, likely depends on a viewer's dispositional FoMO.

Despite this theoretical plausibility, the literature has several limitations. First, conceptual fragmentation is common: studies often treat participation behaviors and psychological outcomes as isolated variables rather than as components of a sequential mechanism. Second, few studies distinguish social presence from social connectedness or test them as a sequential pathway linking specific behaviors to wellbeing. Third, many studies assume a linear positive link between participation and benefits, paying less attention to how anxiety-related traits such as FoMO may condition that link. Synthesizing these gaps, the present study addresses (a) an overemphasis on the bright side of live streaming that neglects a quantity–quality paradox; (b) the absence of integrated serial mediation models testing the transition from social presence to social connectedness; and (c) the rare treatment of FoMO as a moderating boundary condition that alters the quality of the usage experience. Accordingly, we ask two research questions:

- Research Question 1: How is live-streaming participation associated with subjective wellbeing through the sequential mechanisms of social presence and social connectedness?
- Research Question 2: How does trait fear of missing out moderate the pathway between participation and social presence and the subsequent relational mechanisms?

To address these questions, we adopt a quantitative design based on a moderated serial mediation framework, using a cross-sectional online survey of active viewers of chat-based live-streaming platforms. Estimating a regression-based moderated serial mediation model (conceptual Model 85), we test a serial pathway proceeding conceptually from participation to social presence, then to social connectedness, and finally to subjective wellbeing, while modeling FoMO as a moderator of the participation-based paths. We acknowledge that cross-sectional data cannot establish causality; the analysis clarifies the associative pathways through which digital interaction relates to user outcomes and shows how dispositional anxiety may systematically condition the psychological correlates of participation.

This study makes three contributions. First, it addresses conceptual fragmentation by integrating behavioral participation, psychological mechanisms, and emotional outcomes within a single associative framework. Second, it tests social presence and social connectedness as sequential mediators, modeling the pathway from situational experience to internalized connection. Third, by introducing FoMO as a boundary condition, it challenges the assumption that more is better in digital participation and offers a more balanced account of how anxiety shapes the live-streaming experience.

## Literature review and hypotheses development

### Active live streaming participation and subjective wellbeing

Driven by advances in information and communication technologies, live streaming has become a highly accessible and synchronized social-media format (Kang et al., 2021; Zhou et al., 2019) and an important avenue for relaxation and social interaction (Lu et al., 2018). Studies typically operationalize participation through behavioral indicators such as viewing duration, interaction frequency, and virtual gift-giving (Lin et al., 2021). Grounded in uses-and-gratifications theory, researchers have identified diverse motivations for participation, including social interaction, community belonging, tension release, and vicarious experience (Cai and Wohn, 2019; Hilvert-Bruce et al., 2018; Sjöblom and Hamari, 2017), and environmental stimuli such as streamer attractiveness stimulate engagement (Xu et al., 2021).

Historically, participation has been viewed through a commercial lens emphasizing platform stickiness, loyalty, and economic returns (Hu and Chaudhry, 2020; Johnson and Woodcock, 2019; Xi et al., 2023). Although such work acknowledges immersion and interactivity (Li and Peng, 2021), it rarely examines participation as a psychological process related to mental health, and equating high participation with stickiness fails to capture the emotional reconstructions viewers undergo during interaction (Finsterwalder, 2021). From a psychological perspective, active participation represents continuous behavioral investment that may relate to subjective wellbeing. According to self-determination theory, wellbeing relies on the satisfaction of needs for autonomy, competence, and relatedness (Deci et al., 2017; Ryan and Deci, 2000). Unlike passive consumption, active participation—via real-time feedback and expressive interaction—transforms viewers into co-constructors of the digital context, supporting autonomy and competence (Li and Guo, 2021), while synchronous interactivity allows streams to function as temporary communities providing emotional companionship that correlates negatively with loneliness and positively with resilience (Goh et al., 2021; Goh and Tandoc, 2024; Wu, 2022).

The mode of participation is critical. Passive viewing is more weakly associated with positive psychological states than active online interaction such as commenting and responding (Burke and Kraut, 2016; Wolff and Shen, 2024), and active engagement strengthens perceived social support and fosters positive evaluations of the interaction (Meier and Reinecke, 2021). Consistent with this view, recent studies of active sharing on Chinese social-media platforms show that interactive social grooming behaviors are positively associated with well-being and with enjoyment through the accumulation of social capital and social support (Wang and Wang, 2025a,b), underscoring that the benefits of online participation are carried by the relational resources it generates. Active participation may therefore function as a daily regulatory behavior positively associated with life satisfaction. Even so, the active–passive distinction itself has been critiqued as too coarse: a recent person-centered analysis argues that variable-centered active–passive measures emphasize the sheer quantity of use over the qualitative, strategic ways people present themselves, and shows that users cluster into classes, defined jointly by activity and self-presentational selectivity, that differ systematically in personality (Pillow et al., 2025). This implies that the wellbeing correlates of participation may depend in part on who is participating, motivating attention to dispositional moderators of the participation–wellbeing association.

***Hypothesis 1: Active live-stream participation is positively associated with viewers'*** ***subjective wellbeing*.**

### Social presence

Social presence is widely regarded as a defining feature of live-streaming media (Xue et al., 2020). It refers to the degree to which users perceive themselves as connecting with others as real individuals within a mediated environment (Ogara et al., 2014) and is often used to explain cognitive and emotional responses during online interaction, particularly in social commerce, where it enhances trust and continued behavioral intentions (Choi, 2016; Nadeem et al., 2020). Beyond instrumental outcomes, social presence correlates with hedonic benefits and a stronger sense of community belonging (Gao et al., 2017; Weidlich and Bastiaens, 2019).

Social presence theory emphasizes the perception of others during mediated communication (Biocca et al., 2003; Lowry et al., 2009), with perceived presence as the fundamental prerequisite (Lee, 2004). In live streaming, social presence is an experiential state triggered by multidirectional interaction and closely tied to the community's emotional atmosphere (Chen and Lin, 2018). Whereas, prior work often examines system-level antecedents such as interface design (Kim et al., 2020) or gift visualization (Yu et al., 2018), the behavioral act of participation also warrants examination. Viewers express attitudes instantly via bullet screens or virtual gifts, functioning as active co-constructors (Hamari and Sjöblom, 2017). When they receive immediate responses and influence ongoing interaction, their perception of others' real presence is theoretically strengthened (Biocca et al., 2003).

***Hypothesis 2: Active live streaming participation is positively associated with viewers'*** ***social presence*.**

As a core psychological experience, social presence is theorized to relate directly to wellbeing. Experiencing strong social presence fosters positive attitudes toward online communities and a sense of belonging (Gao et al., 2017; Weidlich and Bastiaens, 2019); these elements—pleasant experience, social trust, and belonging—are central components of subjective wellbeing.

***Hypothesis 3: Social presence is positively associated with viewers' subjective*** ***wellbeing*.**

### Social connectedness

Social connectedness is an internalized relational resource reflecting an individual's subjective perception of close, positive relationships within the social world (Grieve et al., 2013; Seppala et al., 2013). According to the belongingness hypothesis, humans possess an innate drive to establish stable, meaningful relationships whose satisfaction is a prerequisite for psychological health (Baumeister and Leary, 1995).

In online settings, social presence is theorized to precede social connectedness. Whereas, social presence captures the immediate, situational feeling of being there with others (Choi, 2016), social connectedness represents a more stable evaluation of social embeddedness and closeness (Gao et al., 2017). Social presence improves the emotional quality of online interaction (Weidlich and Bastiaens, 2019), helping individuals build trust and shorten psychological distance (Zhou et al., 2019); the accumulation of such interactions supports relational continuity and a deeper sense of connectedness (Nadeem et al., 2020).

***Hypothesis 4: Social presence is positively associated with viewers' social*** ***connectedness*.**

Active participation may also provide structural opportunities for developing connectedness. Social media and live-streaming platforms function as networks that facilitate social capital accumulation (Appel et al., 2020; Steinfield et al., 2008), and active online interaction maintains social networks and acquires emotional support (Putnam, 2001), whereas a lack of participation often correlates with isolation (Cacioppo et al., 2015).

***Hypothesis 5: Active live streaming participation is positively associated with viewers'*** ***social connectedness*.**

Social connectedness may function as a proximal correlate of wellbeing. Self-determination theory argues that wellbeing depends on satisfying relatedness needs (Deci et al., 2017); when participation translates into predictable relational ties, this satisfaction forms a foundation for mental health. Higher interaction quality and stronger social orientation predict elevated wellbeing (Reis et al., 2018).

***Hypothesis 6: Social connectedness is positively associated with viewers' subjective*** ***wellbeing*.**

Synthesizing these frameworks, we derive an integrated serial pathway. The model is assembled by chaining three theoretically established links into a single sequence rather than treating them as separate correlations. Self-determination theory specifies relatedness as a proximal driver of wellbeing, which motivates connectedness as the relational resource closest to the outcome (H6); social presence theory specifies perceived co-presence as the experiential precondition from which relational closeness is built, locating presence upstream of connectedness (H4); and research on active vs. passive use locates behavioral participation as the situational trigger of that co-presence (H2). Reading these links in sequence yields a transition from a momentary, situational perception (social presence) to an internalized, dispositional evaluation (social connectedness) and finally to subjective wellbeing. The model is intended to explain not whether participation relates to wellbeing in the aggregate, but through which ordered relational stages that association unfolds. Accordingly, social presence and social connectedness are proposed to operate as serial mediators.

***Hypothesis 7: Social presence and social connectedness play a serial mediating role in*** ***the association between live streaming participation and subjective wellbeing*.**

### Fear of missing out

Because chat-based live streaming is continuous and lacks clear termination cues, trait FoMO represents a crucial boundary condition. FoMO is a stable tendency characterized by persistent anxiety that others are having rewarding experiences from which one is absent (Przybylski et al., 2013), encompassing negative emotion, social comparison, and an impulse to monitor social environments (Vanden Abeele and van Rooij, 2016). From a basic-needs perspective, FoMO is tied to a frustrated need to belong (Deci et al., 2017), and it is consistently correlated with problematic media use, depression, and lower wellbeing, suggesting that increased participation does not automatically yield benefits for highly anxious individuals (Baker et al., 2016; Brown and Kuss, 2020; Deniz, 2021; Elhai et al., 2020). Notably, FoMO has also been shown to condition the relational benefits of live-stream participation itself: among international viewers, the negative association between live interactive singing and loneliness was weaker at higher levels of FoMO (Lin and Wang, 2026), suggesting that anxious monitoring blunts the relational payoff of engagement in the live-streaming context specifically.

We selected trait FoMO, rather than neighboring dispositional constructs such as rejection sensitivity or trait neuroticism, for theoretical reasons specific to this environment. Rejection sensitivity concerns anxious expectations of interpersonal rejection within identified relationships (Downey and Feldman, 1996), and neuroticism indexes a broad tendency toward negative affectivity that shows robust direct associations with lower wellbeing across contexts (Anglim et al., 2020). FoMO is conceptually narrower and more ecologically matched to chat-based streaming: it concerns missing ongoing, diffuse experiences that others are presumed to be enjoying, and it is specifically activated by open-ended environments that offer continuous, never-complete streams of social information. Because chat-based streams have no natural stopping point, they are precisely the kind of setting in which FoMO, as opposed to relationship-specific rejection anxiety or generalized negative affectivity, should govern whether continued participation is experienced as rewarding or as vigilant monitoring. This conceptual specificity also yields a distinct empirical prediction: unlike neuroticism, which would be expected to depress wellbeing directly, FoMO is theorized here to operate as a moderator of the value of participation rather than as a direct determinant of flourishing. We return to this distinction when interpreting the results.

Applying this reasoning, FoMO is theorized to negatively moderate three participation-based paths. First, at the direct level, individuals high in FoMO may participate out of compulsive checking rather than autonomous motivation (Gugushvili et al., 2020), weakening the link between behavioral investment and wellbeing.

***Hypothesis 8: The fear of missing out negatively moderates the direct association*** ***between active live streaming participation and subjective wellbeing*.**

Second, forming social presence requires immersive processing of interactive cues. For individuals high in FoMO, continuous vigilance about potential exclusion occupies limited cognitive resources, disrupting cue integration and attenuating the participation–presence link (Scott and Woods, 2018).

***Hypothesis 9: The fear of missing out negatively moderates the positive association*** ***between active live streaming participation and social presence*.**

Finally, at the relational level, wellbeing depends on whether interaction precipitates into stable, trusting connectedness. When high participation is sustained in an anxious, monitoring state, its association with meaningful ties may be reduced (Wolniewicz et al., 2018).

***Hypothesis 10: The fear of missing out negatively moderates the positive association*** ***between active live streaming participation and social connectedness*.**

**Figure 1:**
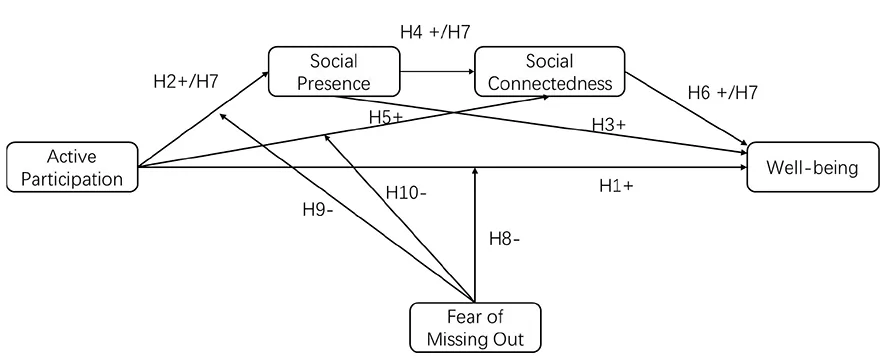
Theoretical model.

Figure 1 presents the theoretical model of this study.

## Methods

### Participants and procedure

This study used a cross-sectional online survey of adult live-stream viewers in China. Ethical approval was obtained from the Institutional Review Board of the School of Journalism and Communication, Sichuan International Studies University (approval No. SISU-JC-2025-041). Participants were recruited and data collected by a professional media market-research company (Hangzhou, Zhejiang, China). Eligible participants were Mandarin-speaking adults (aged 18 years or older) who reported regularly watching live-stream content during the past month. To ensure relevance, respondents confirmed through screening items that they had engaged in at least one interactive behavior (e.g., commenting, reacting, or sending interactive symbols) while watching live streams in the previous month and that they followed at least one streamer they watched regularly.

All participants provided informed consent and were told that participation was voluntary, that responses were anonymous, and that data would be used exclusively for academic research. No personally identifiable information was collected. Attention-check items were embedded, and responses with excessive missing data, patterned responding, or unrealistically short completion times were removed before analysis. The final valid sample comprised *N* = 467 respondents.

Among participants, 45.61% were male and 54.39% were female; mean age was 25.03 years (SD = 5.33, range 18–41). For education, 5.14% had completed high school or below, 14.13% held junior-college qualifications, 56.53% held bachelor's degrees, and 24.20% held postgraduate degrees. Regarding employment, 49.46% were students, 41.11% were employed full-time, 5.57% were freelancers or part-time workers, and 3.85% were otherwise categorized. For relationship status, 63.81% were single, 29.76% were in a relationship or married, and 6.42% were divorced, widowed, or other. Participants were distributed across city tiers in China. As discussed in the Limitations, this recruitment strategy yields a sample selected for active engagement and skewed toward younger, more educated, urban viewers, which bounds generalizability.

Regarding viewing behavior, participants reported a median weekly viewing time of 9.00 hr (M = 12.47, SD = 12.15) and a median viewing history of 23.00 months (*M* = 23.26, SD = 11.69); the median cumulative donation was 390.00 CNY (*M* = 600.63, SD = 597.02). Participants preferred medium-sized channels (37.47%), followed by large (33.19%) and small (29.34%) channels, and primary platforms were distributed across Douyin (26.55%), Bilibili (25.27%), Kuaishou (24.41%), and other platforms (23.77%).

Because participants were Mandarin speakers, the questionnaire was administered in Mandarin. Instruments originally developed in English were translated using a standard forward–backward procedure: two bilingual researchers independently translated and back-translated the items, and discrepancies were resolved through discussion.

### Measurements

All constructs were measured with established Likert-type scales; higher mean scores indicate higher levels of the corresponding construct, and composite indices were computed as item means. To address a reviewer's request, representative items are reported for each scale below, and the contextual framing of each instrument is made explicit. As indicated, the active-participation and social-presence items were tailored to the live-streaming context, whereas social connectedness, fear of missing out, and wellbeing were assessed with the constructs' standard general or dispositional framing, consistent with their conceptualization as relatively global or trait-level constructs. Only the social connectedness scale contained reverse-worded items; the fear-of-missing-out scale contained no reverse-scored items.

Active participation was measured with a three-item scale adapted from the Passive–Active Use Measure (Gerson et al., 2017), reworded to reflect interactive behaviors during live streaming (e.g., I comment directly to the streamer during live streaming; I send emojis, symbols, bullet comments, or other interactive messages to express myself during live streaming). Responses used a 5-point scale (1 = never, 5 = very frequently). The scale showed satisfactory reliability (α = 0.84, *M* = 3.59, SD = 0.81), and exploratory factor analysis supported a single factor (KMO = 0.73, explained variance = 75.48%).

Social presence was measured with a three-item scale adapted from Gefen and Straub (2004) and Sun et al. (2019), assessing perceived interpersonal connection during live streaming (e.g., While watching live streams, I can feel a real sense of social interaction; While watching live streams, I can feel human warmth). Responses used a 5-point agreement scale. Reliability was acceptable (α = 0.76, *M* = 3.48, SD = 0.85; KMO = 0.69, explained variance = 67.32%).

Social connectedness was assessed with the eight-item Social Connectedness Scale–Revised (Lee et al., 2001). Because all items are negatively worded (e.g., Even around other people, I feel alone; I feel left out), all eight items were reverse-coded prior to analysis so that higher values reflect stronger connectedness. Responses used a 5-point agreement scale. Reliability was satisfactory (α = 0.90, *M* = 3.46, SD = 0.83; KMO = 0.94, explained variance = 58.67%).

Trait fear of missing out was measured with five items selected from the original Fear of Missing Out Scale developed by Przybylski et al. (2013). The items assess persistent concerns that others or friends may be having more rewarding experiences, anxiety about not knowing what friends are doing, and distress over missing planned social activities (e.g., I fear others have more rewarding experiences than me; When I miss out on a planned get-together, it bothers me). The items were translated into Mandarin using the forward–backward translation procedure described above. We also refer to Li et al. (2020) as evidence that FoMO can be reliably assessed in Chinese samples, although the present study used a shortened item set based on Przybylski et al.'s (2013) original FoMO framework rather than the full Chinese Trait-State Fear of Missing Out Scale. All items are scored in the same direction; none are reverse-scored. Responses were measured on a 5-point agreement scale. Reliability was good (α = 0.88, *M* = 2.93, SD = 0.82; KMO = 0.89, explained variance = 68.42%).

Wellbeing was measured with a 13-item wellbeing index adapted from the Mental Health Continuum–Short Form (Keyes et al., 2008), which assesses emotional, social, and psychological wellbeing, that is, the positive, flourishing continuum of mental health (Keyes, 2005), over the past month (e.g., I felt happy; I felt that my life had a sense of direction or meaning). Responses used a 5-point frequency scale (1 = never, 5 = every day), and all items were averaged. Reliability was strong (α = 0.94, *M* = 3.75, SD = 0.73; KMO = 0.97, explained variance = 56.79%). Because the instrument indexes positive functioning rather than psychological distress, this measurement choice is relevant to interpreting the FoMO associations reported below.

To reduce potential confounding in the cross-sectional design, the following demographic and behavioral variables were included as covariates in all regression models: gender, age, education, socioeconomic status, marital status, employment status, weekly viewing time, months of live-streaming use, cumulative donation amount, preferred channel size, and primary platform.

### Analysis

All analyses were conducted in Python (version 3.11) using the pandas and numpy libraries for data handling, statsmodels for regression and diagnostics, semopy for confirmatory factor analysis, and factor_analyzer for sampling-adequacy tests. The complete analysis script is provided with the data so that every result reported below can be reproduced exactly. Descriptive statistics and bivariate correlations were computed for all variables. Prior to hypothesis testing, all continuous predictors and the moderator (participation, social presence, social connectedness, and FoMO) were mean-centered to reduce multicollinearity in the interaction terms. Skewness and kurtosis were within acceptable bounds (all |skewness| < 3, all |kurtosis| < 10), supporting parametric analysis.

As an initial diagnostic for common method bias, Harman's single-factor test was conducted; an unrotated principal-components analysis of all 32 items indicated that the first factor accounted for 34.36% of the variance, below the 50% threshold (Podsakoff et al., 2003). Because all focal constructs were self-reported in a cross-sectional design, common method bias cannot be definitively ruled out.

The hypothesized moderated serial mediation model corresponds to Hayes's (2022) conceptual Model 85, in which two mediators operate in sequence and a moderator is specified on the direct path and on the two participation-based paths originating at the predictor. Rather than relying on a packaged macro, we implemented the model directly as a system of ordinary-least-squares path regressions estimated with statsmodels: social presence was regressed on participation, FoMO, and their product; social connectedness on participation, social presence, FoMO, and the participation × FoMO product; and wellbeing on participation, social presence, social connectedness, FoMO, and the product. The 11 covariates were entered into all three equations, and heteroscedasticity-consistent (HC3) standard errors were used throughout. Conditional (simple-slope) associations were evaluated at the mean and at ±1 SD of FoMO using the model variance–covariance matrix. Indirect effects (the two single-mediator paths and the serial path), evaluated at the mean of the moderator, were tested with 5,000 bootstrap resamples (a fixed random seed makes the resampling exactly reproducible), and an effect was considered statistically significant when its 95% percentile confidence interval (CI) excluded zero. To probe the interactions beyond the pick-a-point (±1 SD) approach, we additionally computed Johnson–Neyman regions of significance, which identify the precise values of the moderator at which the conditional association of participation transitions into and out of statistical significance (Bauer and Curran, 2005; Preacher et al., 2006).

## Results

### Measurement quality and common method bias

Harman's single-factor test indicated that the first unrotated factor accounted for 34.36% of the variance, below the 50% threshold (Podsakoff et al., 2003), suggesting that no single factor dominated the covariance among items; nonetheless, because all constructs were self-reported, common method bias cannot be fully excluded. A confirmatory factor analysis on the full measurement model indicated excellent fit, χ^2^/df = 1.05, RMSEA = 0.010, SRMR = 0.028, CFI = 0.997, TLI = 0.997. Composite reliabilities ranged from 0.76 to 0.94, and average variance extracted (AVE) ranged from 0.51 to 0.63, supporting convergent validity. Discriminant validity was established using the Fornell–Larcker criterion: the square root of the AVE for each construct exceeded its correlations with the others (Table 1).

**Table 1:** Discriminant validity (Fornell–Larcker criterion) and intercorrelations.

Variable	1	2	3	4	5
1. Active participation	0.80				
2. Social presence	0.37^**^	0.71			
3. Social connectedness	0.44^**^	0.45^**^	0.73		
4. Fear of missing out	−0.03	−0.00	0.01	0.78	
5. Subjective wellbeing	0.44^**^	0.50^**^	0.57^**^	0.03	0.73

Diagonal elements (in bold conceptually) are the square root of the AVE; off-diagonal elements are Pearson correlations.

^**^p < 0.01.

### Direct and mediation pathways

**Figure 2:**
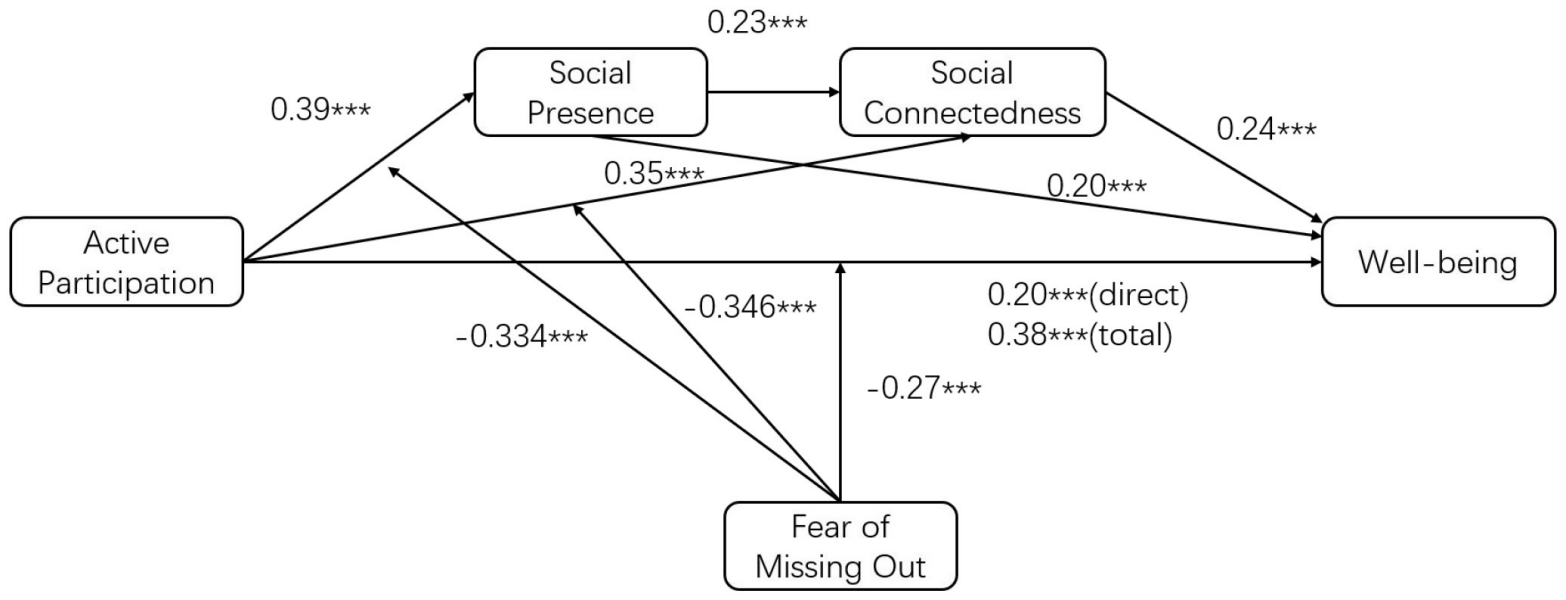
Path coefficients. ***p < 0.001.

Consistent with H1, active participation was positively associated with subjective wellbeing, *b* = 0.20, SE = 0.04, *t* = 5.36, *p* < 0.001, 95% CI [0.13, 0.27]. Active participation was positively associated with social presence (H2), *b* = 0.39, SE = 0.05, *t* = 8.46, *p* < 0.001, 95% CI [0.30, 0.48], and social presence was positively associated with wellbeing (H3), *b* = 0.20, SE = 0.03, *t* = 5.96, *p* < 0.001, 95% CI [0.14, 0.27]. Social presence was positively associated with social connectedness (H4), *b* = 0.23, SE = 0.04, *t* = 5.28, *p* < 0.001, 95% CI [0.15, 0.32], and participation was positively associated with connectedness (H5), *b* = 0.35, SE = 0.04, *t* = 8.30, *p* < 0.001, 95% CI [0.27, 0.43]. Finally, connectedness was positively associated with wellbeing (H6), *b* = 0.24, SE = 0.04, *t* = 6.07, *p* < 0.001, 95% CI [0.16, 0.32]. Thus, H1–H6 were supported (Table 2). Figure 2 summarizes the estimated model with path coefficients (detailed in Appendix).

**Table 2:** Regression results for direct and mediation pathways.

Pathway	*b*	SE	*t*	*p*	95% CI
Active participation → Social presence	0.39	0.05	8.46	< 0.001	[0.30, 0.48]
Social presence → Social connectedness	0.23	0.04	5.28	< 0.001	[0.15, 0.32]
Social connectedness → Subjective wellbeing	0.24	0.04	6.07	< 0.001	[0.16, 0.32]
Active participation → Subjective wellbeing	0.20	0.04	5.36	< 0.001	[0.13, 0.27]
Indirect: Participation → Presence → Wellbeing	0.08	0.02	—	—	[0.05, 0.11]
Indirect: Participation → Connectedness → Wellbeing	0.08	0.02	—	—	[0.05, 0.12]
Indirect: Participation → Presence → Connectedness → Wellbeing	0.02	0.01	—	—	[0.01, 0.04]

Unstandardized coefficients with 11 covariates controlled. Indirect effects are percentile bootstrap CIs (5,000 resamples) from the path regression model (conceptual Model 85), evaluated at the mean of the moderator.

### Serial mediation

The serial indirect association from participation through social presence to social connectedness to wellbeing was statistically different from zero at the mean level of FoMO, indirect effect = 0.02, 95% CI [0.01, 0.04]. Two parallel single-mediator pathways were also significant: through social presence alone, 0.08, 95% CI [0.05, 0.11], and through social connectedness alone, 0.08, 95% CI [0.05, 0.12]. H7 was therefore supported, although the serial indirect effect was modest in magnitude.

### Moderation by fear of missing out

**Figure 3:**
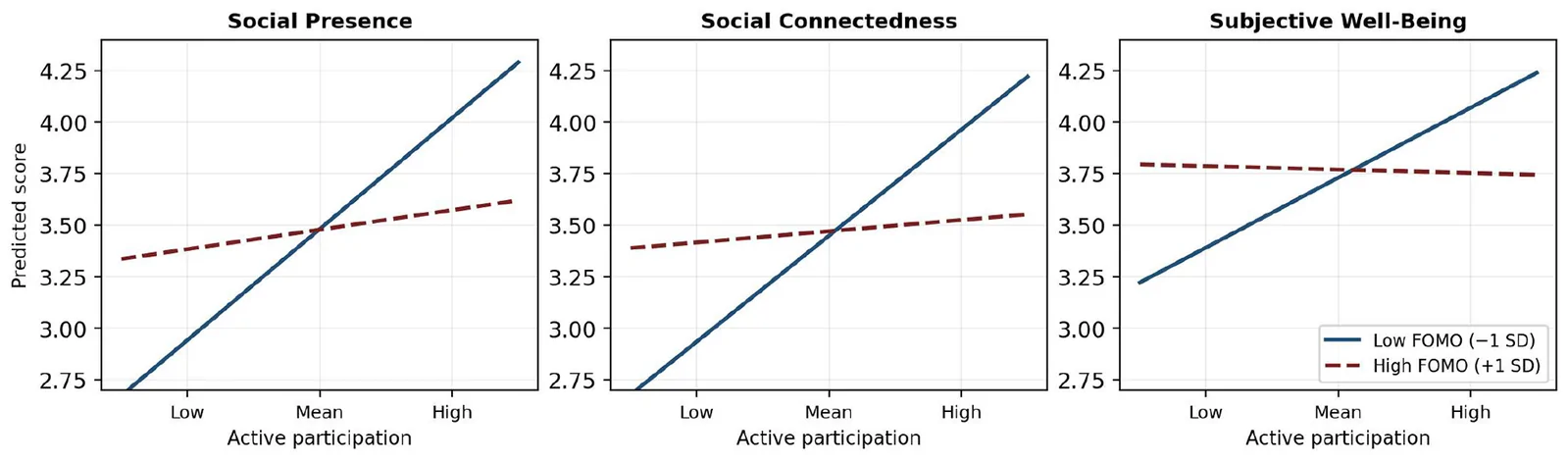
Simple slopes of active participation at low vs. High fear of missing out. Predicted scores for active participation at low (−1 SD) vs. high (+1 SD) fear of missing out. Lines cross near the sample mean of participation, consistent with a near-zero main effect of fear of missing out.

As shown in Table 3, the FoMO main effect was non-significant in every model (all ps > 0.45), whereas the Participation × FoMO interaction was significantly negative for social presence (*b* = −0.33, SE = 0.06, *p* < 0.001), social connectedness (*b* = −0.35, SE = 0.05, *p* < 0.001), and wellbeing (*b* = −0.27, SE = 0.04, *p* < 0.001). The interaction accounted for a non-trivial increment in explained variance (Δ*R*^2^ = 0.067, 0.069, and 0.048, respectively). Simple-slope analyses (Table 4; Figure 3) showed the expected crossover pattern: at low FoMO (−1 SD), participation was strongly and positively associated with wellbeing (*b* = 0.42, *p* < 0.001), social presence (*b* = 0.66, *p* < 0.001), and connectedness (*b* = 0.64, *p* < 0.001), whereas at high FoMO (+1 SD) these associations were weak and non-significant. H8–H10 were supported.

**Table 3:** Moderating associations of fear of missing out.

Variable	Model 1 (Presence)	Model 2 (Connectedness)	Model 3 (Wellbeing)
Constant	3.63^***^	3.22^***^	3.64^***^
Active participation	0.39^***^	0.35^***^	0.20^***^
Social presence	—	0.23^***^	0.20^***^
Social connectedness	—	—	0.24^***^
Fear of missing out	0.00	0.01	0.02
Participation × FoMO	−0.33^***^	−0.35^***^	−0.27^***^
*R* ^2^	0.23	0.38	0.49
Δ*R*^2^ (interaction)	0.067	0.069	0.048

Unstandardized coefficients; 11 covariates controlled; HC3 standard errors.

^***^p < 0.001.

**Table 4:** Conditional associations of participation at different levels of fear of missing out.

Outcome	FoMO level	*b*	SE	*t*	*p*
Subjective wellbeing	Low (−1 SD)	0.42	0.05	7.74	< 0.001
	Mean	0.20	0.04	5.36	< 0.001
	High (+1 SD)	−0.02	0.04	−0.51	0.614
Social presence	Low (−1 SD)	0.66	0.06	10.75	< 0.001
	Mean	0.39	0.05	8.46	< 0.001
	High (+1 SD)	0.12	0.07	1.69	0.092
Social connectedness	Low (−1 SD)	0.64	0.06	10.48	< 0.001
	Mean	0.35	0.04	8.30	< 0.001
	High (+1 SD)	0.07	0.06	1.08	0.279

Conditional effects of active participation estimated at the mean and ±1 SD of fear of missing out.

### Probing the interactions: Johnson–Neyman regions of significance

Because the pick-a-point approach evaluates the conditional association at only three arbitrary values of the moderator, and because a reviewer raised the question of whether a strong interaction can coexist with a negligible main effect, we probed the interactions using the Johnson–Neyman technique (Bauer and Curran, 2005; Preacher et al., 2006). This procedure identifies the exact values of FoMO at which the conditional association of participation crosses the threshold of statistical significance, and therefore provides a more complete picture than the ±1 SD slopes.

**Figure 4:**
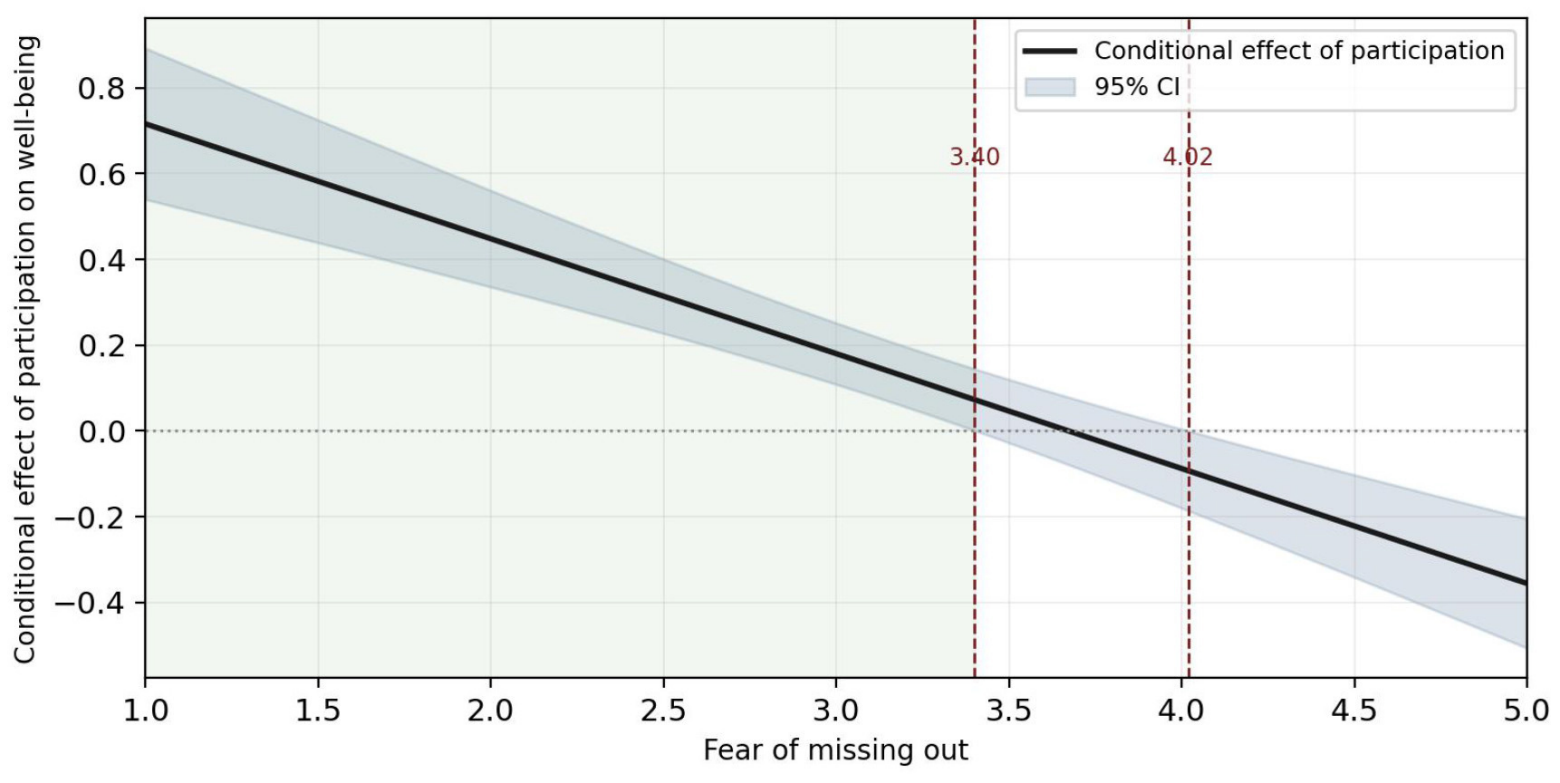
Johnson–Neyman plot: region of significance for wellbeing. The solid line is the conditional association of active participation with subjective wellbeing across levels of fear of missing out; the shaded band is the 95% confidence interval. The association is significant where the band excludes zero. Vertical lines mark the Johnson–Neyman points (3.40 and 4.02).

For subjective wellbeing, the association between participation and wellbeing was significantly positive for viewers below the lower Johnson–Neyman point of 3.40 (74.7% of the sample), non-significant between 3.40 and 4.02 (18.4% of the sample), and significantly negative above 4.02 (6.9% of the sample). The same pattern held for the relational mediators: the participation association was significantly positive below FoMO values of 3.70 (social presence) and 3.62 (social connectedness), covering 81.4% of the sample in each case, non-significant across the next interval, and significantly negative only at the extreme upper tail (above 4.82 for social presence and 4.49 for social connectedness, i.e., fewer than 3% of cases). Thus, the positive association between participation and the model outcomes held across most of the observed range of FoMO and weakened, ultimately reversing, only at the upper extreme of the distribution. Figure 4 displays the region of significance for wellbeing. These results are summarized in Table 5.

**Table 5:** Johnson–Neyman regions of significance for the conditional association of participation.

Outcome	Lower J–N point	Upper J–N point	% sig. positive	% non-significant	% sig. negative
Subjective wellbeing	3.40	4.02	74.7%	18.4%	6.9%
Social presence	3.70	4.82	81.4%	18.2%	0.4%
Social connectedness	3.62	4.49	81.4%	16.3%	2.4%

FoMO was measured on a 1–5 scale (M = 2.93, SD = 0.82).

The lower and upper Johnson–Neyman points are the values of FoMO at which the 95% confidence interval for the conditional association of active participation includes zero; the association is significantly positive below the lower point, non-significant between the two points, and significantly negative above the upper point.

Percentages are the share of the sample in each region.

Two features of the FoMO findings warrant emphasis because they bear on the interpretation developed in the Discussion. First, the near-zero association between FoMO and the model variables is not an artifact of measurement direction: the FoMO scale contains no reverse-scored items, all five items loaded positively on a single factor, and the scale showed strong internal consistency (α = 0.88). Second, the FoMO–wellbeing association showed no evidence of curvilinearity: a quadratic FoMO term added to the wellbeing model was non-significant (*b* = −0.01, SE = 0.03, *p* = 0.75, 95% CI [−0.08, 0.06]). Together with the near-zero linear association, this is consistent with FoMO relating to flourishing not directly but, in this dataset, primarily as a boundary condition on the value of participation.

### Model diagnostics and robustness

Variance inflation factors indicated no multicollinearity concern (maximum VIF = 1.57), and Breusch–Pagan tests indicated acceptable homoscedasticity (all robust ps > 0.05); nonetheless, HC3 standard errors were retained as a conservative specification. The model was estimated with and without the 11 covariates; the magnitude and significance of all hypothesized paths, including the direct associations, the serial mediation, and the FoMO moderation, were robust across specifications. In the wellbeing model, only socioeconomic status showed a small positive association (*b* = 0.05, *p* = 0.044); other covariates were non-significant. Unmeasured confounding cannot be excluded.

## Discussion

This study examined how active participation in chat-based live streaming is associated with subjective wellbeing, and clarified the relational mechanisms and dispositional boundary conditions underlying that association. Drawing on self-determination theory and social presence theory, a moderated serial mediation model linked participation to wellbeing through social presence and social connectedness, with trait FoMO moderating the participation-based pathways. Three findings stand out: participation was positively associated with wellbeing directly and through both relational mediators; the serial pathway from situational co-presence to internalized connectedness was statistically detectable but small; and trait FoMO conditioned the value of participation while showing essentially no direct association with flourishing.

### Situating the findings in prior research

The positive association between active participation and wellbeing aligns closely with the communication literature distinguishing active from passive use. Meta-analytic and conceptual reviews indicate that interactive, expressive use of social media is more consistently linked to positive psychological states than passive consumption (Burke and Kraut, 2016; Meier and Reinecke, 2021), and the present results extend this active-use advantage to the chat-based streaming context, converging with work showing that livestream viewing can buffer loneliness and support wellbeing (Goh et al., 2021; Goh and Tandoc, 2024; Wolff and Shen, 2024). The pattern is particularly close to recent evidence that a specific live-stream behavior, live interactive singing, is negatively associated with loneliness through social connection and social support among international viewers (Lin and Wang, 2026), reinforcing the view that participation in live streaming relates to wellbeing by way of the relational resources it cultivates. The mediating roles of social presence and connectedness are likewise consistent with social presence theory (Biocca et al., 2003; Lee, 2004) and with belonging-based accounts of online relationship formation (Gao et al., 2017; Nadeem et al., 2020), and they parallel findings that active online sharing relates to wellbeing through accumulated social capital (Wang and Wang, 2025a), supporting the view that perceived co-presence is an experiential precursor to more stable relational closeness (Roth and Peng, 2024).

At the same time, the results diverge from prior work in two informative ways. First, the serial indirect effect, although significant, was small (0.02), and the single-mediator indirect effects were themselves modest (0.08). This is a divergence in magnitude rather than direction: studies that frame relational mediation as a central explanatory mechanism may overstate its incremental contribution to global wellbeing. The modest size is plausible because flourishing is shaped by many influences beyond a single media behavior, and it reinforces a cautious reading in which chat-based participation functions as a supplementary, not a primary, relational resource. Second, and more notably, trait FoMO was essentially unrelated to wellbeing at the bivariate level (*r* = 0.03), which appears to depart from a literature that frequently reports negative FoMO–wellbeing associations (Baker et al., 2016; Elhai et al., 2020; Przybylski et al., 2013). We address this apparent discrepancy directly in the next section, because it is central to interpreting the role of FoMO in this model.

### Reconciling the role of fear of missing out

Three considerations help reconcile the negligible direct association between FoMO and wellbeing with the broader literature, and together they reframe the apparent anomaly as a theoretically coherent result. First, and most importantly, the discrepancy is partly a matter of which continuum of mental health is being measured. Contemporary models treat positive mental health (flourishing) and psychological distress (illbeing) as related but distinct dimensions rather than opposite poles of one continuum (Keyes, 2005). The dependent measure here, the Mental Health Continuum–Short Form, indexes the flourishing continuum (emotional, psychological, and social wellbeing), whereas the reliable negative correlates of FoMO in prior work are concentrated on the distress continuum: depression, anxiety, negative affect, and problematic use (Elhai et al., 2020; Wolniewicz et al., 2018). The dependent measure here, the Mental Health Continuum–Short Form, indexes the flourishing continuum (emotional, psychological, and social wellbeing), whereas the reliable negative correlates of FoMO in prior work are concentrated on the distress continuum: depression, anxiety, negative affect, and problematic use (Elhai et al., 2020; Wolniewicz et al., 2018). Prior studies cited by the reviewer demonstrate that FoMO is often linked to wellbeing-related outcomes, but they differ from the present study in design, focal outcome, and the modeled role of FoMO. Brown and Kuss (2020), for example, used a seven-day social media abstinence design and found that abstinence was accompanied by increased mental wellbeing and social connectedness and decreased FoMO. Deniz (2021) modeled FoMO as a mediator between social self-efficacy and life satisfaction. In contrast, the present study examined FoMO as a moderator of participation-based pathways in an already-engaged live-streaming sample and used a flourishing-oriented wellbeing index. Thus, our near-zero bivariate association should not be read as contradicting these studies, but as suggesting that FoMO may operate differently when modeled as a boundary condition rather than as a direct predictor or mediator. Second, the sample was selected for active engagement and skews young, educated, and urban, which may attenuate direct FoMO effects relative to broader or more distressed populations. A near-zero FoMO–flourishing association is therefore considerably more consistent with the literature than a generic FoMO–wellbeing framing would suggest, and it is precisely what the two-continua model would predict. Second, the sample was selected for active engagement and skews young, educated, and urban, which may attenuate direct FoMO effects relative to broader or more distressed populations. Third, FoMO was assessed as a stable trait; trait FoMO need not depress average mood even where momentary, state FoMO does.

Crucially, the negligible main effect coexisted with robust moderation, and this combination is not a statistical contradiction but a pattern consistent with a pure-moderation interpretation. A predictor with no main effect but a strong interaction does not shift the average level of the outcome; instead, it changes the slope relating another predictor to that outcome. Substantively, FoMO did not make viewers less happy on average, it changed whether their participation paid off relationally. The crossover form visible in Figure 3, in which the low- and high-FoMO lines intersect near the participation mean, is exactly what a near-zero moderator main effect produces, and the Johnson–Neyman analysis shows that the protective association of participation held for roughly three quarters to four fifths of the sample, fading only at the upper tail of FoMO. Read this way, FoMO is best understood not as a direct determinant of flourishing but as a boundary condition on the quality of participation, consistent with our a priori rationale for preferring FoMO over neuroticism, which would have been expected to depress wellbeing directly. This reading is consistent with FoMO operating primarily as a boundary condition in this dataset rather than as a form of general negative affectivity; however, this interpretation remains inferential because alternative dispositional moderators such as neuroticism and rejection sensitivity were not measured.

### Theoretical contributions

The study offers three contributions. First, it addresses conceptual and methodological fragmentation by integrating behavioral participation, interaction-based psychological experience, and a wellbeing outcome within a single moderated serial mediation framework, linking specific situational behavior to broader psychological states rather than treating them as isolated correlations. Second, it supports a meaningful and empirically validated distinction between social presence and social connectedness; discriminant-validity results justify treating them as distinct rather than interchangeable, with presence capturing an immediate, situational sense of being with others and connectedness representing a more internalized evaluation of belonging. Third, by positioning FoMO as a moderator rather than an antecedent of use frequency, the study provides evidence consistent with a quantity–quality paradox: the same participatory behaviors are less positively associated with psychological outcomes for highly anxious individuals. This conclusion converges with person-centered accounts of social-media use, which argue that the active–passive distinction is too coarse and that variable-centered measures can obscure person × environment interactions bearing on wellbeing: a recent HEXACO-based typology found that user classes defined jointly by activity and self-presentational selectivity differ systematically in personality, most notably extraversion and emotionality (Pillow et al., 2025). Our findings extend this person-centered logic to live streaming by showing that a single disposition, trait FoMO, conditions whether active participation is associated with flourishing. The pattern documented here, strong conditioning of the participation slope alongside no direct association with flourishing, consistent with FoMO operating primarily as a boundary condition in this dataset, adds nuance to FoMO theory by showing that, against a positive-functioning outcome, FoMO may matter chiefly as a regulator of the value of engagement.

### Practical implications

For platforms, the results suggest that designing solely to maximize participation metrics such as chat volume may be insufficient for supporting wellbeing, because the psychological value of participation depends on a viewer's dispositional anxiety. Although specific design features were not tested, the moderation pattern is consistent with the possibility that interfaces encouraging continuous monitoring, persistent prompts or notifications that discourage natural stopping points, could amplify FoMO-related dynamics; design research might examine whether interfaces that support healthy stopping cues are associated with more positive experiences than those emphasizing continuous vigilance. For streamers and community managers, the mediation results indicate that participation relates to wellbeing primarily when it fosters genuine social presence and connectedness, suggesting that community norms emphasizing warmth and reciprocal acknowledgment may be more beneficial than norms encouraging competition for the streamer's attention. For users and practitioners, the conditional pattern implies that participation is most likely to be beneficial when experienced as voluntary and relationally meaningful rather than as anxious monitoring; digital wellbeing education could help users distinguish supportive engagement from engagement driven by fear of missing out.

### Limitations and future research

Several limitations qualify these findings. First, the design was cross-sectional, so the sequential mediation model rests on theoretical assumptions and supports no causal or temporal claims. Reverse causality is plausible, viewers with higher baseline wellbeing or connectedness may participate more actively, as is a third-variable account in which a stable personality profile drives both active participation and the cultivation of supportive relationships and wellbeing. Recent typological work indicates that distinct user profiles in social-media ecosystems are systematically associated with different personality configurations (Pillow et al., 2025), raising the possibility that the observed associations partly reflect selection of certain dispositions into active participation rather than effects of participation itself. Future research should use longitudinal cross-lagged designs, experience-sampling, or person-centered methods to model how organic participation co-varies with wellbeing over time and to help disentangle these accounts.

Second, although demographic and behavioral covariates were included, omitted-variable bias remains. In particular, the dataset did not include alternative dispositional moderators such as trait neuroticism, rejection sensitivity, baseline loneliness, social anxiety, or trait positive affect, so we could not test directly whether such traits show the same crossover pattern as FoMO or account for the present associations. This is a meaningful constraint on the claim that FoMO operates distinctly from general negative affectivity; that claim is supported here by theory and by the pattern consistent with pure moderation, but it remains inferential and a direct empirical test awaits a study that measures these neighboring constructs alongside FoMO. We regard such a study as the most valuable next step.

Third, the sample was selected for active engagement (interaction within the past month and following at least one regular streamer) and skews young, highly educated, urban, and culturally homogeneous (Chinese viewers). Consequently, the participation associations are estimated within an already-engaged population and may not generalize to casual or passive viewers or to other cultural contexts; replication in more heterogeneous samples is warranted. Fourth, participation was operationalized as generalized frequency, which does not capture qualitative differences (e.g., supportive conversation vs. performative emote spamming); multidimensional measures could test whether different forms of participation yield divergent outcomes. Fifth, although the dependent measure indexes the flourishing continuum, future work that includes distress-oriented outcomes (e.g., anxiety, problematic use) alongside flourishing would allow a more complete test of how FoMO relates to both continua of mental health within the live-streaming context. Finally, the present study is exclusively quantitative; future mixed-methods designs that pair survey data with qualitative accounts of viewers' lived experience could illuminate the meaning of participation and the subjective texture of FoMO-driven monitoring, strengthening the explanation of the mechanisms inferred here.

## Conclusion

Using a moderated serial mediation model, this cross-sectional study found that active participation in chat-based live streaming was positively associated with subjective wellbeing, both directly and through a sequence proceeding from social presence to social connectedness. Trait fear of missing out functioned as a boundary condition: although it was essentially unrelated to flourishing at the bivariate level, it robustly conditioned the value of participation, such that the positive association held for most viewers and weakened, ultimately reversing for wellbeing, only at the upper extreme of FoMO. These findings articulate a psychological paradox of digital environments: high behavioral engagement does not automatically yield wellbeing, particularly when participation may be driven by anxiety rather than autonomous motivation. By integrating behavioral, relational, and emotional dimensions within a theory-informed framework, the study advances understanding of both the relational potential and the dispositional constraints of contemporary live-streaming engagement.

## Data Availability

The raw data supporting the conclusions of this article will be made available by the authors, without undue reservation.
