# Supplementary material for: The impact of active live-stream participation on wellbeing: the mediating roles of social presence and connectedness and the moderating role of fear of missing out
Source: Front Psychol. 2026 Aug 21;17:1824481. doi: 10.3389/fpsyg.2026.1824481 (PMC13543407; doi:10.3389/fpsyg.2026.1824481)
Supplement: Supplementary file 1 [file Supplementary_file_1.docx]

Supplementary Material

# 1 Appendix A. Path Coefficient Report

This appendix reports the coefficient and significance for every path in the estimated moderated serial mediation model. Variables: Active Participation (ALP), Social Presence (SP), Social Connectedness (SCS), Subjective Well-Being (WB); moderator: Fear of Missing Out (FOMO). N = 467; OLS path regressions (Hayes Model 85) with HC3 standard errors; 5,000 seeded bootstrap resamples; 11 covariates entered in every equation; focal predictors mean-centered; all coefficients are unstandardized (b). Estimates reproduce exactly from livestream_analysis.py (seed = 20240517).

## 1.1 Direct structural paths (estimated at mean FOMO)

| **Path** | **Label** | **b** | **SE** | **t** | **p** | **95% CI** | **Sig.** |
| --- | --- | --- | --- | --- | --- | --- | --- |
| ALP → SP | a1 | +0.391 | 0.046 | +8.46 | < .001 | [0.300, 0.481] | *** |
| ALP → SCS | a2 | +0.351 | 0.042 | +8.30 | < .001 | [0.268, 0.434] | *** |
| SP → SCS | d | +0.234 | 0.044 | +5.28 | < .001 | [0.147, 0.321] | *** |
| SP → WB | b1 | +0.205 | 0.034 | +5.96 | < .001 | [0.137, 0.272] | *** |
| SCS → WB | b2 | +0.239 | 0.039 | +6.07 | < .001 | [0.162, 0.316] | *** |
| ALP → WB (direct) | c′ | +0.199 | 0.037 | +5.36 | < .001 | [0.126, 0.272] | *** |
| ALP → WB (total) | c | +0.384 | 0.036 | +10.76 | < .001 | [0.314, 0.454] | *** |

*Note. All six structural paths are positive and significant at p < .001. The total effect (c) equals the direct effect (c′) plus the total indirect effect: 0.199 + 0.186 = 0.385, which matches c = 0.384 within rounding. *** p < .001.*

## 1.2 Fear of missing out: main effects and interaction (moderation) terms

| **Term** | **Outcome** | **b** | **SE** | **t** | **p** | **95% CI** | **Sig.** |
| --- | --- | --- | --- | --- | --- | --- | --- |
| FOMO (main effect) | SP | −0.002 | 0.047 | −0.04 | .968 | [−0.094, 0.090] | ns |
| FOMO (main effect) | SCS | +0.013 | 0.040 | +0.31 | .754 | [−0.066, 0.091] | ns |
| FOMO (main effect) | WB | +0.024 | 0.032 | +0.74 | .462 | [−0.039, 0.086] | ns |
| ALP × FOMO | SP | −0.334 | 0.057 | −5.84 | < .001 | [−0.446, −0.222] | *** |
| ALP × FOMO | SCS | −0.346 | 0.054 | −6.37 | < .001 | [−0.453, −0.240] | *** |
| ALP × FOMO | WB | −0.268 | 0.038 | −7.14 | < .001 | [−0.342, −0.195] | *** |

*Note. FOMO has no significant main effect on any variable, but significantly and negatively moderates all three participation-origin paths. Interaction ΔR² = .067 (SP), .069 (SCS), and .048 (WB). *** p < .001; ns = not significant. SP = social presence; SCS = social connectedness; WB = well-being.*

## 1.3 Conditional effects of participation (simple slopes by FOMO level)

| **Outcome** | **Low FOMO (−1 SD)** | **Mean FOMO** | **High FOMO (+1 SD)** |
| --- | --- | --- | --- |
| Subjective well-being | +0.419*** | +0.199*** | −0.021 (p = .614) |
| Social presence | +0.664*** | +0.391*** | +0.117 (p = .092) |
| Social connectedness | +0.635*** | +0.351*** | +0.068 (p = .279) |

*Note. Entries are the simple slope of active participation on each outcome (b) at −1 SD, the mean, and +1 SD of FOMO. The positive association of participation weakens as FOMO rises and is non-significant at high FOMO. *** p < .001.*

## 1.4 Johnson–Neyman regions of significance

| **Outcome** | **Significant positive** | **Non-significant** | **Significant negative** |
| --- | --- | --- | --- |
| Subjective well-being | FOMO < 3.40 (74.7%) | 3.40–4.02 (18.4%) | FOMO > 4.02 (6.9%) |
| Social presence | FOMO < 3.70 (81.4%) | 3.70–4.82 (18.2%) | FOMO > 4.82 (0.4%) |
| Social connectedness | FOMO < 3.62 (81.4%) | 3.62–4.49 (16.3%) | FOMO > 4.49 (2.4%) |

*Note. FOMO is on its raw 1–5 metric; percentages are the share of the sample in each region. The association of participation is significantly positive across most of the FOMO range and turns non-significant (and, for a small upper tail, negative) only at high FOMO.*

## 1.5 Indirect effects (5,000 bootstrap resamples, at mean FOMO)

| **Indirect path** | **Effect (b)** | **95% bootstrap CI** | **Sig.** |
| --- | --- | --- | --- |
| ALP → SP → WB | +0.080 | [0.051, 0.113] | Yes |
| ALP → SCS → WB | +0.084 | [0.053, 0.118] | Yes |
| ALP → SP → SCS → WB (serial) | +0.022 | [0.012, 0.035] | Yes |
| Total indirect | +0.186 | [0.142, 0.231] | Yes |

*Note. Indirect effects use 5,000 percentile bootstrap resamples evaluated at the mean of FOMO. An effect is significant when the 95% CI excludes zero; all four do.*

## 1.6 Model fit and robustness

| **Equation (outcome)** | **R²** | **Adjusted R²** | **Interaction ΔR²** |
| --- | --- | --- | --- |
| Social presence | .233 | .199 | .067 |
| Social connectedness | .377 | .347 | .069 |
| Subjective well-being | .489 | .464 | .048 |

*Note. Measurement model: CFA χ²/df = 1.05, CFI = TLI = .997, RMSEA = .010, SRMR = .028; reliabilities α = .84 (ALP), .76 (SP), .90 (SCS), .88 (FOMO), .94 (WB).*

Robustness checks. (a) With versus without the 11 covariates: the sign and significance of every hypothesized path are unchanged (e.g., a1 = .39 with covariates vs .38 without; all three interactions remain p < .001). (b) Curvilinearity: a quadratic FOMO term added to the well-being model is non-significant (b = −.01, SE = .03, p = .75, 95% CI [−.08, .06]). (c) Diagnostics: maximum VIF = 1.57 (no multicollinearity concern), and Breusch–Pagan tests are non-significant for all three models (all ps > .05); HC3 standard errors were retained as a conservative specification.

*Verification. A re-run of livestream_analysis.py on livestream_data.csv reproduced every value above exactly (the bootstrap is seeded); the full console output is saved in analysis_output.txt.*

# 2 Appendix B. Variable Definitions and Descriptive Statistics

This appendix reports the full names, abbreviations, model roles, and coding schemes of all study variables (Table A1), construct-level descriptive statistics and internal consistency estimates (Table A2), and item-level descriptive statistics with full item wording for every measurement item (Table A3). All values were computed using the same reproducible Python pipeline used for the analyses reported in the main text (N = 467).

**Table A1**

*Study Variables: Full Names, Abbreviations, Model Roles, and Coding*

| **Variable** | **Abbreviation** | **Role in model** | **Coding / scale** |
| --- | --- | --- | --- |
| Active live-stream participation | ALP | Predictor (X) | 3 items; 1–5 Likert-type scale |
| Social presence | SP | Mediator (M1) | 3 items; 1–5 Likert-type scale |
| Social connectedness | SCS | Mediator (M2) | 8 items; reverse-coded; 1–5 Likert-type scale |
| Fear of missing out | FOMO | Moderator (W) | 5 items; 1–5 Likert-type scale |
| Subjective well-being | WB | Outcome (Y) | 13 items; 1–5 frequency scale |
| Gender | GENDER | Covariate | 1 = male, 2 = female |
| Age | AGE | Covariate | Years |
| Education | EDU | Covariate | 1 = high school or below; 2 = junior college; 3 = bachelor’s degree; 4 = postgraduate degree or above |
| Socioeconomic status | SES | Covariate | Personal monthly income level: 1 = 3,000 CNY or below; 2 = 3,001–5,000 CNY; 3 = 5,001–8,000 CNY; 4 = 8,001–15,000 CNY; 5 = above 15,000 CNY |
| Marital status | MARITAL | Covariate | 1 = single/unmarried; 2 = in a relationship/married; 3 = divorced/widowed/other |
| Employment status | EMP | Covariate | 1 = student; 2 = full-time employed; 3 = freelancer/part-time; 4 = other |
| Weekly viewing time | TIMEW | Covariate | Hours per week |
| Months of live-streaming use | MONTHS | Covariate | Months |
| Cumulative donation | DONATE | Covariate | CNY |
| Preferred channel size | CHANNEL | Covariate | 1 = small; 2 = medium; 3 = large |
| Primary platform | PLATFORM | Covariate | 1 = Douyin; 2 = Kuaishou; 3 = Bilibili; 4 = other |

*Note. ALP, SP, SCS, FOMO, and WB were measured using 5-point scales. Composite scores were computed as item means. The eight social connectedness items were reverse-coded before computing the SCS composite so that higher scores indicate stronger social connectedness. WB denotes the 13-item well-being index adapted from the Mental Health Continuum–Short Form. The item codes MHC1–MHC13 refer to the 13 well-being items used to compute WB.*

**Table A2**

*Construct-Level Descriptive Statistics and Internal Consistency*

| **Construct** | **Abbrev.** | **k** | **M** | **SD** | **α** | **Observed range** |
| --- | --- | --- | --- | --- | --- | --- |
| Active participation | ALP | 3 | 3.59 | 0.81 | 0.84 | 1.00–5.00 |
| Social presence | SP | 3 | 3.48 | 0.85 | 0.76 | 1.00–5.00 |
| Social connectedness | SCS | 8 | 3.46 | 0.83 | 0.90 | 1.00–5.00 |
| Fear of missing out | FOMO | 5 | 2.93 | 0.82 | 0.88 | 1.00–5.00 |
| Subjective well-being | WB | 13 | 3.75 | 0.73 | 0.94 | 1.08–5.00 |

*Note. N = 467. k = number of items; M and SD are for the composite item mean; α = Cronbach’s alpha; observed range is the minimum–maximum of the composite score.*

**Table A3**

*Item-Level Descriptive Statistics and Item Wording*

| **Construct** | **Item** | **Item wording** | **M** | **SD** | **Skew** | **Kurt** | **r** |
| --- | --- | --- | --- | --- | --- | --- | --- |
| Active participation | ALP1 | I comment directly to the streamer during live streaming. | 3.59 | 0.92 | −0.26 | −0.54 | 0.68 |
|  | ALP2 | I respond to the streamer or other viewers during live streaming. | 3.58 | 0.93 | −0.13 | −0.56 | 0.71 |
|  | ALP3 | I send emojis, symbols, bullet comments, or other interactive messages to express myself during live streaming. | 3.60 | 0.94 | −0.28 | −0.42 | 0.71 |
| Social presence | SP1 | While watching live streams, I can feel a sense of contact with the streamer or other viewers. | 3.48 | 1.06 | −0.33 | −0.49 | 0.61 |
|  | SP2 | While watching live streams, I can feel a real sense of social interaction. | 3.49 | 1.04 | −0.39 | −0.30 | 0.57 |
|  | SP3 | While watching live streams, I can feel human warmth. | 3.48 | 1.00 | −0.28 | −0.46 | 0.59 |
| Social connectedness | SCS1 | I feel disconnected from the world around me. | 3.45 | 1.10 | −0.26 | −0.64 | 0.68 |
|  | SCS2 | Even around other people, I feel alone. | 3.45 | 1.11 | −0.32 | −0.67 | 0.64 |
|  | SCS3 | I feel distant from people. | 3.47 | 1.06 | −0.30 | −0.50 | 0.74 |
|  | SCS4 | I have no sense of belonging. | 3.45 | 1.07 | −0.27 | −0.58 | 0.69 |
|  | SCS5 | I feel that people are around me but not with me. | 3.48 | 1.09 | −0.37 | −0.48 | 0.67 |
|  | SCS6 | I feel left out. | 3.50 | 1.07 | −0.28 | −0.66 | 0.70 |
|  | SCS7 | I do not feel related to anyone. | 3.47 | 1.08 | −0.29 | −0.67 | 0.64 |
|  | SCS8 | I feel isolated from others. | 3.43 | 1.06 | −0.43 | −0.43 | 0.69 |
| Fear of missing out | FOMO1 | I fear others have more rewarding experiences than me | 2.99 | 1.06 | −0.14 | −0.61 | 0.70 |
|  | FOMO2 | I fear that my friends have more rewarding experiences than me. | 2.93 | 0.96 | −0.10 | −0.37 | 0.76 |
|  | FOMO3 | I get worried when I find out that my friends are having fun without me. | 2.87 | 0.98 | +0.07 | −0.35 | 0.69 |
|  | FOMO4 | I get anxious when I do not know what my friends are up to. | 2.92 | 0.98 | −0.01 | −0.35 | 0.70 |
|  | FOMO5 | When I miss out on a planned get-together or activity, it bothers me. | 2.95 | 0.98 | −0.02 | −0.52 | 0.75 |
| Subjective well-being | MHC1 | I felt happy. | 3.75 | 0.98 | −0.45 | −0.33 | 0.70 |
|  | MHC2 | I felt interested in life. | 3.72 | 0.96 | −0.49 | −0.25 | 0.76 |
|  | MHC3 | I felt satisfied with life. | 3.74 | 0.98 | −0.55 | −0.12 | 0.72 |
|  | MHC4 | I felt that I belonged to a community or social group. | 3.76 | 0.93 | −0.47 | −0.10 | 0.70 |
|  | MHC5 | I felt that society makes sense. | 3.72 | 0.98 | −0.58 | −0.05 | 0.73 |
|  | MHC6 | I felt that I had something important to contribute to society. | 3.79 | 0.95 | −0.44 | −0.45 | 0.61 |
|  | MHC7 | I felt that society is becoming a better place for people. | 3.75 | 1.01 | −0.58 | −0.20 | 0.69 |
|  | MHC8 | I felt that people are basically good. | 3.77 | 0.99 | −0.55 | −0.14 | 0.70 |
|  | MHC9 | I liked most parts of my personality. | 3.76 | 0.92 | −0.30 | −0.51 | 0.65 |
|  | MHC10 | I was good at managing the responsibilities of my daily life. | 3.71 | 0.98 | −0.46 | −0.26 | 0.74 |
|  | MHC11 | I had warm and trusting relationships with others. | 3.76 | 0.96 | −0.44 | −0.24 | 0.68 |
|  | MHC12 | I felt that my life had a sense of direction or meaning. | 3.68 | 0.96 | −0.39 | −0.22 | 0.73 |
|  | MHC13 | I felt that I was growing as a person. | 3.79 | 0.98 | −0.70 | +0.24 | 0.74 |

*Note. N = 467. Skew = skewness; Kurt = kurtosis; r = corrected item–total correlation. For SCS1–SCS8, the table displays the original negatively worded item wording, whereas the item-level descriptive statistics and corrected item–total correlations are based on the reverse-coded values used in the analysis; thus, higher item scores indicate stronger social connectedness after reverse-coding. The FOMO items are all keyed in the same direction and were not reverse-coded. MHC1–MHC13 are the 13 items used to compute the WB composite, a well-being index adapted from the Mental Health Continuum–Short Form. All items fall within conventional limits for skewness and kurtosis (|skewness| < 1, |kurtosis| < 1).*

# 3 Composite Score Calculation

```
ALP  = mean(ALP1, ALP2, ALP3)
SP   = mean(SP1, SP2, SP3)
rSCS1–rSCS8 = 6 − SCS1–SCS8
SCS  = mean(rSCS1, rSCS2, rSCS3, rSCS4, rSCS5, rSCS6, rSCS7, rSCS8)
FOMO = mean(FOMO1, FOMO2, FOMO3, FOMO4, FOMO5)
WB   = mean(MHC1, MHC2, MHC3, MHC4, MHC5, MHC6, MHC7, MHC8, MHC9, MHC10, MHC11, MHC12, MHC13)
```

In the moderated serial mediation model, ALP was specified as X, SP as M1, SCS as M2, FOMO as W, and WB as Y.
